# Development of tau targeted protein degraders for Alzheimer disease

**DOI:** 10.1002/alz.086380

**Published:** 2025-01-09

**Authors:** Lisha Wang, Rajnish Kumar, Pavel F. Pavlov, Bengt Winblad

**Affiliations:** ^1^ Department of Neurobiology, Care Sciences and Society, Division of Neurogeriatrics, Karolinska Institutet, Solna Sweden; ^2^ Department of Pharmaceutical Engineering & Technology, Indian Institute of Technology, Varanasi India; ^3^ Theme Inflammation and Aging, Karolinska University Hospital, Stockholm Sweden; ^4^ Division of Neurogeriatrics, Karolinska Institutet, Stockholm Sweden

## Abstract

**Background:**

Alzheimer disease (AD) is a progressive neurodegenerative disease that is accountable for the leading case of dementia in elder people. Before, only symptomatic treatments are available for AD. Since 2021, two anti‐amyloid antibodies aducanumab and lecanemab have been approved by the US Food and Drug Administration. However, controversies are still around these two antibodies regarding their efficacy, safety, and substantial economic burden. They also have restricted brain delivery and could not pass through intact neurons to target intracellular proteins. There is still an urgent need for effective, easily administered (oral) and low‐cost drugs for AD.

The intraneuronal accumulation of tau paired helical filaments (PHFs) is one of the hallmarks of AD brain pathology. Lowered efficiency of degradation pathways, such as ubiquitin proteasome system, further exacerbates tau pathologies. Instead of inhibiting protein aggregation, a class of molecules that promote the degradation of targeted protein is known as proteolysis‐targeting chimeras (PROTACs). Since 2016, PROTACs have been applied to resolve tau pathologies, progressing from being based on peptides to fully synthetic small molecules. This exploration is still at the early stage. Here, we aim to develop novel small‐molecule PROTACs as tau targeted protein degraders for AD treatment.

**Methods:**

We performed global molecular docking of tau binders from a public database (bindingdb.org) into the available PHFs 3D structure and selected top ranked compounds. Together with them, ligands binding to E3 ligase, for instance cereblon (CRBN) and carboxyl terminus of Hsp70 interacting protein (CHIP), were used with different linkers to design synthetically accessible PROTACs. The modelling of ternary complex (tau‐PROTACs‐E3 ligase) are performed to rank designed PROTACs and the highly scored will be synthesised. Protein binding affinities and tau‐reducing effects will be evaluated in different cell models: human neuroblastoma SH‐SY5Y cells and mouse embryonic primary neurons.

**Results:**

We perform the development of tau targeted protein degraders according to the methods listed above. We selected the top ranked tau binders and designed new PROTACs.

**Conclusions:**

The most potent PROTACs with least off‐target effects will move on to *in vivo* study and provide pre‐clinical evidence for novel treatment of AD tauopathies.

